# The impact of alternative arrangements, contingent jobs, and work secured through an app on the well-being of working age adults: Results from the California Work and Health Survey

**DOI:** 10.1002/ajim.23625

**Published:** 2024-06-17

**Authors:** Edward Yelin, Laura Trupin, Trisha Iley, Nari Rhee, Alicia Lafrance, Ima Varghese Mac

**Affiliations:** 1 Philip R Lee Institute for Health Policy Studies, UCSF, San Francisco, California, USA; 2 Center for Labor Research and Education, UC, Berkeley, California, USA

**Keywords:** alternative employment arrangements, appbased work, contingent employment, impacts on economic and health status and working conditions

## Abstract

**Background:**

There is recognition of the growing prevalence of alternative work arrangements, contingent jobs, and work secured through an app. However, there have been few systematic efforts to understand the impact of these forms of work on individuals and households.

**Methods:**

The data derive from the California Work and Health Survey administered to a sample of the working age population of the state solicited through random-digit dialing of cell phone numbers. 4014 individuals completed the survey, 26% of those with an in-service cell phone number. We present odds ratios and 95% confidence intervals from logistic regression estimating the impact of being an independent contractor, in other forms of alternative work arrangements, in contingent jobs, and in work secured through an app, on economic and health status and working conditions in main jobs, with and without adjustment for covariates.

**Results:**

Several of the forms of work analyzed are associated with lower earnings and higher rates of wage theft, household poverty, benefit recipiency, and expectation of hardships in food, housing, and medical care in the immediate future. Association between the forms of work and current health status is less consistent. However, several forms of work are associated with working conditions known to be risk factors for subsequent health problems.

**Conclusions:**

Public policy to mitigate the adverse impacts of work, largely developed in the 20th Century when there was an identified workplace, may be insufficient to protect workers’ well-being for alternative work arrangements, contingent jobs, and work secured through an app.

## 1 | INTRODUCTION

The impact of forms of work, spanning alternative work arrangements, contingent jobs, and work secured through an app have become an important focus of public concern.^1^ Part of the concern has been about the magnitude of these forms, specifically whether they are growing at the expense of more traditional employment in which the worker is formally hired by the firm for which the work is done on a more permanent basis.^2^ Part, too, is due to the growing visibility of task- and project-based work secured through apps that connect workers to customers.^3,4^

While trends in the magnitude of alternative work arrangements are subject to debate,^5–8^ another issue is the impact of these arrangements on the well-being of the workers and their families in a range of domains, including economic sufficiency and worker health and safety.^1,9–14^ The lack of a permanent relationship between workers and firms also severs one avenue of career mobility, the internal labor market of the firm itself. The effects of alternative work arrangements, contingent jobs, and work secured through an app may also burden government at all levels tasked with ensuring access to safe work - difficult when there is no on-going relationship between worker and firm- often with no identified workplace; providing a safety net of health insurance through such mechanisms as Medicaid and the Affordable Care Act; and providing income support through such mechanisms as the Earned Income Tax Credit and the Supplemental Nutrition Assistance Program. Indeed, more than almost any other nation, our system of income and health security is tied to the provision of benefits through employment. This system may not serve workers who are not formally or permanently hired to do work.^15^

The use of alternative work arrangements, contingent jobs, and work secured through an app is increasingly understood in the context of larger corporate strategies to reduce exposure of the firm to the cost of labor, labeled the *fissuring of work*,^16^ and to shift the risks to workers and their families.^17^ The use of these overarching strategies may grow even if the use of some of their specific aspects, like the use of alternative work arrangements, does not.

The present study is designed to provide estimates of the impact of several forms of work, including alternative ways of hiring, contingent jobs, and app-based work, on economic well-being of individuals and households, their health status, and on-the-job exposures.

## 2 | METHODS

The data presented below are from the California Work and Health Survey (CWHS). The CWHS is designed to provide a comprehensive picture of the employment situation and health status of a random sample of the working age (18–70 years) population of the state,. Although it emulates the methods and many of the measures of the Census Bureau’s Contingent and Alternative Work Supplement to the Current Population Survey,^18^ it expands the scope of the labor market data to include both main and secondary jobs and the time-frame of data capture to incorporate both the week and month before interview, reflecting suggestions for improving estimates of these forms of employment.^5^ Sampling for the CWHS was accomplished by random digit dialing from listings of contract and pre-paid cell phone numbers. After contact was made, respondents could choose to be interviewed by a survey interviewer or complete an online version. Both interviewer and self-administered versions were available in English and Spanish. Data collection was completed between November 2022 and May 2023. A total of 4014 persons completed the survey, representing 26% of those successfully contacted. At the conclusion of the data collection, sample adjustment weights were developed^19^ based on the population estimates of demographic characteristic, geography, and socioeconomic status of the population of California adults ages 18–70 from the Census Bureau’s 2021 American Community Survey.^20^ After collecting information on the employment status of respondents in the week and month before interview, the CWHS asked about the presence of alternative, contingent, and app-based work in main and second jobs, working conditions in those jobs, current health status, economic well-being, assessment of economic strain, receipt of public and private benefits, and standard demographic variables. The present study is limited to the effect of characteristics of main jobs.

A methods compendium with greater detail on sampling, weighting, and content of the survey may be found at the CWHS website: calaborlab.ucsf.edu/cwhs.

The data collection protocol, including consent at the time of survey administration, was approved by the UCSF Institutional Review Board.

### 2.1 | Analyses

We use logistic regression to estimate the impact of four forms of employment on economic outcomes, health status, and working conditions in the main job, with and without covariates which may confound the relationship between the nature of work and the outcomes. The four forms include independent contracting, the most common of the alternative ways people are engaged to do work; a variable for the other three kinds of alternative employment (hereafter: “other alt work”) including on-call work, being hired by a temporary agency, and having one’s work subcontracted out; contingent employment, defined as expecting a job held for less than a year to last less than a year into the future; and work secured through an app. Many of the working conditions included as outcomes are known risk factors for subsequent occupational health events, for example, irregular shifts, high levels of ergonomic demands, and jobs with high demands and low levels of control.^21–28^

The covariates in the adjusted models include age, gender, race/ethnicity, marital status, household size, education level, occupations, occupational tenure, industries, and job tenure.

We present odds ratios and 95% confidence intervals for the effect of the four forms of work on the economic outcomes, measures of health status, and each sentinel working condition.

As a sensitivity analysis, we disaggregated independent contractors into two groups, the self-employed and those deemed wage- and salary-independent contractors, by indicating that they worked for someone else as an independent contractor, freelance, or consultant, including but not limited to a business or farm that they own. We also disaggregated other forms of alternative work into on-call workers, those hired by a temp agency, or those reporting their work was subcontracted out. As these results did not differ substantially from the results reported below, to simplify the presentation, we show the results only for the principal analysis using the aggregated versions of independent contractors and other forms of alternative work. The results of the sensitivity analysis are available from the CWHS website: https://calaborlab.ucsf.edu/file/sensitivity-analyses-impact-forms-workxlsx-0.

## 3 | RESULTS

Table 1 shows the employment status of working age Californians (18–70 years of age) and, among those employed, those who are independent contractors, in other forms of alternative work, in contingent jobs, and in app-based work, by demographic characteristics. In the month before survey, 70.8% of working-age Californians were employed. Among the employed (Column 1), 16.6% were independent contractors, 10.7% were in other forms of alternative work, 5.6% were in contingent jobs, and 6.1% reported app-based work. Employment rates peaked among those 30–39, were higher among males, non-Hispanic Whites and Asian or Pacific Islanders, and rose substantially with each increment of educational level.

Columns two through four show the distribution of the forms of work by demographic characteristics. Being an independent contractor was more common among those 65–70 and among persons with lower levels of educational attainment. In contrast, other forms of alternative work were more common among younger workers. The latter forms of work were more common among members of minority groups than among non-Hispanic Whites, those who were never married, and among those with a high school education or less. Contingent employment was also more common among younger workers, members of minority groups, and those with lower levels of education. App-based work occurred more frequently among younger workers and was extremely uncommon among the oldest age group of workers and among those with graduate training.

Tables 2–4 show the results of the analysis of the effects of forms of work on economic outcomes, health status, and working conditions, respectively. On an unadjusted basis, all the forms of work analyzed when compared to persons not in these forms are associated with earnings at or below $40,000/year, approximately the estimate of a living wage for a single person with no children in California.^29^ After adjusting for potential confounders, all but those in other alt work also are more likely to have earnings at or below this level (Table 2). Reported wage theft is more likely among those in all forms of work analyzed, with the exception that, on an adjusted basis, those in app-based work are not significantly more likely to report this. As a result of having less access to health insurance through work, those who are independent contractors, in other alt work, and in contingent jobs are less likely to have health insurance when interviewed and at any point in the prior year. For independent contractors, this result holds after adjustment.

Turning to household economic well-being, the second set of rows in Table 2, those in each of the four forms of work analyzed are more likely to have household incomes at or below 125% of the Federal poverty level even though those in independent contracting, other alt work, and contingent employment are more likely to have other sources of income beyond their own earnings. Before adjustment, those in each of the forms of work report difficulty living on their current household income; this was true for all but those in independent contracting after adjustment, too. In dealing with problems in the immediate future, on an unadjusted basis, those in contingent jobs and in work secured through an app report that they cannot sustain an emergency expense of $400, while those in other alt work, in contingent jobs, and app-based work indicate that they expect actual hardships in food, housing, and medical care in the ensuing 2 months after interview. The latter situation is true for those in contingent jobs and app-based work even after adjustment. The CWHS collected information about receipt of several kinds of income transfer programs. With adjustment, those who are in the categories of independent contractors, other alt work, and app-based employment are more likely to receive public or private disability compensation. Independent contractors, contingent workers, and those in app-based employment are also more likely to report receiving Supplemental Nutrition Assistance Program (SNAP) benefits or using a food bank in the year before interview.

The forms of work analyzed are less consistently related to health status than to economic well-being (Table 3). Contingent employment does appear to be related to mental health status, to high levels of perceived stress and to high levels of pain, while forms of alternative work other than independent contracting may be related to experiencing numbness, to high levels of alcohol consumption, and to on-the-job injuries in the year before interview. The long latency period in many symptoms and specific chronic diseases may make it hard to detect health impacts of the forms of work we analyzed, since these kinds of jobs are often held for relatively short tenures.

Long latency in health problems is why epidemiologists often focus on measures of heightened risk for the future development of symptoms and diseases, in the case of occupational health this means the patterning of working conditions. Table 4 portrays the relationship of the forms of work analyzed to working conditions, some, as noted above, with known etiological connections to health status.^21–28^

Several of these forms are proposed as a way of providing flexibility to workers in how they work. The results bear this out, with workers who are independent contractors and those in app-based work more likely to report having flexible work schedules. Having flexible work also may mean having irregular shifts, with those in all forms of work analyzed other than contingent jobs reporting irregular shifts. Similarly, those who are independent contractors and those in app-based employment are more likely to regularly work from home (the latter only after adjustment for covariates). High levels of ergonomic exposure subject workers to risk of musculoskeletal problems.^26^ Independent contractors and those in other alt work are more likely to report high levels of ergonomic demands, as were those in app-based jobs on an unadjusted basis. Despite the trope about white collar work predominating among independent contractors,^30^ they and, before adjustment, persons in other alt work and app-based work are less likely to report high levels of cognitive demands in their work. The combination of high work demands and low levels of control is a well-known risk factor for several diseases.^25–27^ Independent contractors are less likely to report such a risky combination, but those in other alt work and in contingent jobs are more likely to do so.

The CWHS included measures of interactions with coworkers, supervisors, and customers, including bullying and shows of respect. None of the forms of work analyzed are associated with bullying; those in other alt work are less likely to report respectful relationships on the job. On an unadjusted basis, contingent workers do as well. The CWHS queried about perceptions of levels of education necessary to do one’s job. Only those in contingent work report higher levels of education than they deemed necessary to do their jobs and only before adjustment. Both independent contractors and those in contingent jobs are less likely to report that their jobs represented either a promotion within a job or a new, better job. Although answers may not reflect their current jobs, we asked CWHS respondents to report whether they experienced discrimination in employment over their careers, with those in contingent employment more likely to report experiencing it.

**FIGURE 1:**
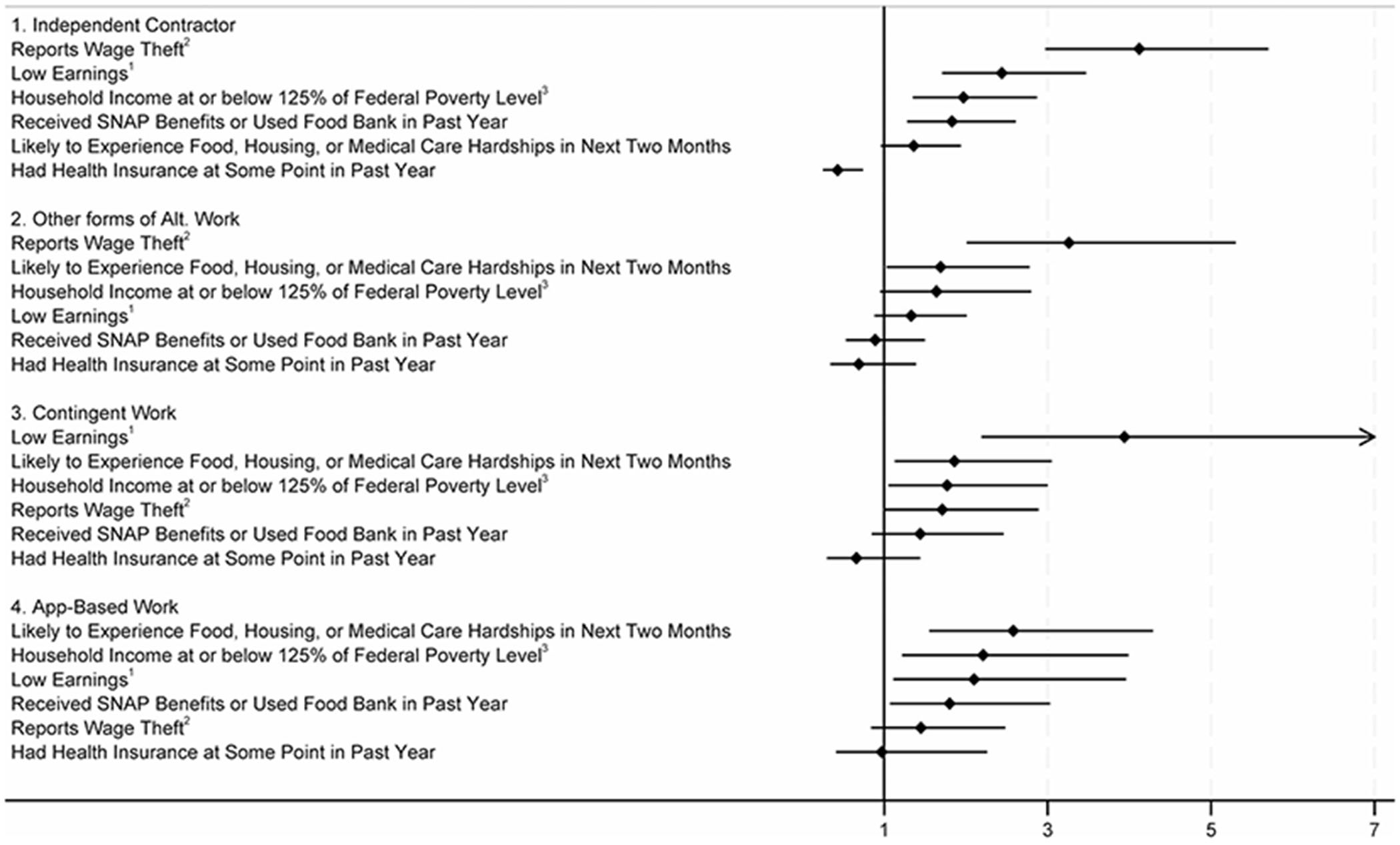
Forest plot of impact of forms of nonstandard employment in main job (odds ratios & 95% confidence intervals) on principal economic outcomes of Californians Employed in Month Before Interview. 1. Earnings at or below $40,000. 2. Wage theft defined as having done work for which pay not received or receiving less pay than expected. 3. Poverty measure defined using combination of household income and household size.

The relationship between the forms of work is most consistent for economic outcomes. Figure 1 uses a forest plot to highlight the magnitude of the effects of the forms of employment for the principal economic measures: earnings, wage theft, household poverty, economic strain, and benefit recipiency. Although the magnitude of the effects vary by the specific form of employment (e.g., the odds of reporting wage theft are greater for independent contractors than those in contingent jobs) each of the forms was associated with at least several of the economic outcomes, highlighting the effects on the individual and household as well as on society due to benefit recipiency.

## 4 | DISCUSSION

Although hardly as comprehensive as in many other developed nations, during the 20th Century the US developed a set of laws and regulations to protect workers from discrimination in hiring, promotion, and retention on the basis of race, ethnicity, gender, and disability status; to help them attain a minimum of economic security while employed through rights to organize, the establishment of a wage floor, and, more recently, mandating employment-based health insurance coverage; minimizing the economic impacts of work loss through unemployment insurance; minimizing the health risks of work through workers’ compensation and occupational health and safety; and maintaining a minimum standard of living in retirement through Social Security and Medicare.^9,14^

This system of protections was developed over roughly a century, starting with workers’ compensation in the first two decades of the last century, and culminating in the passage of the Affordable Care Act in 2010. With certain exceptions, such as the availability of Social Security to self-employed workers, the tie that binds these protections is that they assume that the worker is formally hired by the firm for which work is done. The ride-share driver may not attain a minimum wage when the costs of car ownership are factored in. The person shopping for and then delivering groceries may be at risk for musculoskeletal injuries, just as might the person stocking the shelves in a supermarket who was formally hired to do the work, but without the possibility of filing a workers’ compensation claim and without an Occupational and Health Safety Administration (OSHA) inspection to investigate the cause of the injury. While the system of protections for workers formally hired is hardly ironclad—think about the fate of undocumented workers in dangerous factories—workers who are not formally hired by the firm for whom the work is done have fewer protections. Although those in contingent jobs may be formally hired, they too may experience work exposures that adversely affect them after the job is completed. In California, the site of our study and the focus of the California Labor Laboratory, our Center funded by the National Institute for Occupational Safety and Health (NIOSH), there has been considerable legislative and regulatory ferment to try to mitigate the effects of being an independent contractor, having other forms of alternative work, or work secured through an app, principally through Assembly Bill 5 (AB5) which established legal criteria for determining whether, in fact, someone should be considered an employee rather than outside contractor. The effects of AB5 have been somewhat offset by the passage of Proposition 22 in which ride share and delivery companies sought to delimit the reach of AB5 while providing some protections in its stead.^31^ As of this writing, the constitutionality of Proposition 22 is before the California Supreme Court. At the Federal level, a new regulation also seeks to mitigate the effects of this kind of work through regulation of the definition of independent contracting.^32^

But what are the risks associated with alternative, contingent, and app-based work? The present study used a random sample of working age residents of California to document the effects of these forms of work on economic well-being, current health status, and working conditions, many of which are known risks for later health problems. There were strong associations of earnings at or below a living wage for a single individual and high reported frequency of wage theft. Indeed, on an unadjusted basis, 55% of independent contractors, 47% of those in other forms of alternative employment, 77% of those in contingent jobs, and 67% of those in app-based employment in their main jobs reported low earnings; even after adjustment, the analogous figures were 52%, 41%, 59%, and 52%, respectively in main jobs.

Almost tautologically, workers in alternative arrangements are less likely to have access to a pension or employment-based health insurance. The availability of Medicaid or ACA-related subsidies is insufficient to overcome the lack of employment-based insurance so that, for instance, independent contractors are significantly less likely to have had any health insurance in the year before interview, let alone currently.

Having other members of the household in the labor market is insufficient to overcome the effects of these forms of work on the economic well-being of the household; those in each of these forms of employment in their main jobs are more likely to have household incomes at or below 125 percent of the Federal poverty level. Not surprisingly, they are more likely to report difficulty in living on their current household incomes and those in contingent- or app-based work were more likely to state that they expected actual hardships in food, housing, or medical care in the 2 months subsequent to interview. Indeed, independent contractors, contingent workers, and those in app-based employment are also more likely to have received SNAP benefits or used a food bank in the prior year; contingent workers are also more likely to report being unhoused at the time of interview. All this suggests that beyond the economic impacts on the workers themselves as well as their households, some of the impacts are externalized to society as a whole.

For whatever reason, we did not observe as consistent a set of findings on the relationship of the forms of work analyzed to health status as to economic well-being. This may be because there are no consistent health effects or because of the long latency period for many health problems. To deal with the latter problem, we will be re-interviewing the respondents to the CWHS in another 2 years when health effects may come to light. Nevertheless, we did observe that those in contingent jobs experience a higher probability of reporting fair or poor mental health and high levels of perceived stress. This may indicate that the insecurity inherent in contingent jobs may exact a toll on mental health. It is also noteworthy that persons in other forms of alternative employment are at higher risk of on-the-job injuries, to receive medical treatment for them, and to report them to their employers. These effects may reflect a reduced surveillance of on-call workers, those in temp agency work, or whose labor is subcontracted out because the firm has less attachment to and perhaps investment in the well-being of workers brought on by these mechanisms.

Risk factor analysis of differences in working conditions may alert workers, healthcare providers, and policymakers to problems that may arise in the years to come. Risks identified here with known relationships to future health problems include those associated with irregular shifts, high levels of ergonomic demands, and, for those in several forms of work analyzed, the combination of high demands and low control. On the other hand, higher rates of flexible-hours work may protect independent contractors and app-based workers from some of the effects of the risky working conditions identified.

Although there may be some beneficial working conditions associated with the forms of work we analyze, on balance we identified a panoply of adverse effects, including a higher probability of low earnings and wage theft, poverty-level incomes for the household, certain forms of benefit recipiency, experience of financial hardship at the time of interview and expectation of severe hardship in the months to come.

The strategy of trying to bring more workers into the system of protections for formally-hired workers reflected in legislation like California’s AB5 is an important initial strategy to sustain the well-being of the working age population.^31^ However, it is insufficient because industries that stand to benefit from having more workers put in alternative arrangements, contingent jobs, or task- or project-based work secured through an app have the resources to fight the passage of such legislation or through the initiative process, as was done when they sponsored Proposition 22. It will be difficult to build a system of protections for workers with no permanent connection to a workplace and sometimes with no defined workplace, rendering the workers invisible, but this is not a reason not to try. As shown here, the workers themselves are experiencing the consequence of increases in alternative, contingent, and app-based work, but society at large is paying the costs through a range of programs spanning income transfers, SNAP benefits, provision of subsidized health insurance through the ACA or Medicaid, and the mitigation of health problems that may arise in the years to come. Data like those presented here can spark debate about how we can proceed to protect the welfare of our workforce when the firm no longer hires workers formally or permanently.

## Figures and Tables

**TABLE 1:** Employment status in month before interview and kind of employment in main job, by demographic characteristics of the California working age population.

		Kind of employment among those employed			
	Employed in past month	Independent contractor^1^	Other forms of alternative employment^2^	Contingent work^3^	App-based work^4^
All	70.8%	16.6%	10.7%	5.5%	6.1%

Age					
18–24	66.8%	12.1%	15.8%	17.0%	9.7%
25–29	79.6%	14.7%	12.5%	7.0%	7.5%
30–39	80.2%	13.1%	13.2%	2.9%	7.0%
40–44	76.0%	14.9%	6.7%	5.4%	6.7%
45–49	76.7%	14.7%	8.8%	3.1%	5.2%
50–59	73.1%	20.5%	8.7%	2.1%	3.8%
60–64	58.7%	21.9%	4.8%	3.5%	3.2%
65–70	38.4%	37.3%	7.6%	5.8%	0.6%

Gender					
Male	74.7%	17.1%	12.5%	5.0%	6.0%
Female	66.8%	16.1%	8.5%	6.1%	6.1%

Race/Ethnicity					
White, non-Hispanic	73.1%	17.8%	6.4%	3.7%	4.8%
Hispanic or Latino	66.8%	14.2%	15.1%	6.5%	7.6%
Asian or Pacific Islander	77.6%	12.1%	11.4%	7.8%	6.0%
Black or African American	69.6%	18.9%	10.0%	7.9%	6.1%
Other (includes mixed race & Native American)	70.6%	21.2%	11.2%	5.7%	5.3%

Marital status					
Married	74.3%	15.0%	8.9%	3.5%	3.7%
Widowed, separated, or divorced	59.7%	21.3%	9.5%	5.6%	7.0%
Never Married	70.7%	16.7%	13.8%	8.8%	9.1%

Education level					
High School or Less	60.5%	19.0%	16.0%	6.5%	7.8%
Some College	70.1%	16.3%	9.8%	6.4%	7.8%
Bachelor’s Degree	79.8%	14.5%	7.8%	5.5%	4.1%
Graduate Training	88.6%	14.9%	6.9%	2.3%	1.7%

1 Either self-employed or work for someone else as an independent contractor.

2 Category includes on-call workers, those employed by temp. agency, and those whose work is subcontracted out.

3 Contingent work is defined as a job not expected to last another 12 months.

4 App-based employment defined as jobs or tasks that connect workers to customers via an app.

**TABLE 2:** Impact of independent contracting, other forms of alternative employment, contingent work, and app-based work vs. traditional forms of employment in the main job on economic status of worker and household.

		Independent contractor^1^ Weighted percent: 16.8%		Other forms of alternative employment^2^ Weighted percent: 9.8%		Contingent work^3^ Weighted percent: 5.4%		App-based work^4^ Weighted percent: 5.6%	
Economic status	Weighted % with work condition	Unadjusted	Adjusted	Unadjusted	Adjusted	Unadjusted	Adjusted	Unadjusted	Adjusted
		Cells are odds ratios & 95% confidence intervals for comparison of form of employment vs. Traditional. Cells in bold are statistically significant.							
Worker earnings and benefits									
Low earnings^5^	39.1%	**2.31 (1.77, 3.02)**	**2.44 (1.71, 3.47)**	**1.74 (1.20, 2.53)**	1.33 (0.88, 2.01)	**5.84 (3.75, 9.11)**	**3.94 (2.19, 7.09)**	**4.05 (2.56, 6.42)**	**2.10 (1.11, 3.96)**
Reports wage theft^6^	11.4%	**3.96 (2.82, 5.57)**	**4.12 (2.97, 5.70)**	**3.70 (2.21, 6.18)**	**3.26 (2.01, 5.30)**	**2.13 (1.30, 3.50)**	**1.71 (1.01, 2.89)**	**1.94 (1.16, 3.25)**	1.45 (0.84, 2.48)
Reports bonus or tips as part of compensation	36.3%	1.07 (0.82, 1.40)	1.10 (0.84, 1.46)	0.74 (0.51, 1.08)	0.74 (0.49, 1.14)	**0.58 (0.38, 0.90)**	**0.54 (0.33, 0.88)**	**2.39 (1.57, 3.64)**	**2.54 (1.63, 3.97)**
Has no paid sick days	5.5%	1.16 (0.71, 1.91)	1.00 (0.57, 1.77)	1.09 (0.52, 2.28)	0.86 (0.39, 1.88)	**3.20 (1.49, 6.85)**	**2.51 (1.20, 5.22)**	1.01 (0.46, 2.22)	0.77 (0.31, 1.89)
Has access to pension or tax-deferred savings plan	55.7%	**0.12 (0.09, 0.17)**	**0.11 (0.08, 0.16)**	**0.54 (0.38, 0.77)**	0.71 (0.47, 1.07)	**0.32 (0.21, 0.50)**	**0.47 (0.29, 0.78)**	**0.22 (0.14, 0.36)**	**0.33 (0.19, 0.57)**
Has access to employer health insurance plan	73.7%	**0.12 (0.09, 0.16)**	**0.13 (0.09, 0.17)**	**0.64 (0.43, 0.97)**	0.79 (0.51, 1.23)	**0.39 (0.26, 0.60)**	**0.48 (0.30, 0.78)**	**0.38 (0.25, 0.58)**	**0.47 (0.28, 0.79)**
Currently on employer health insurance plan	52.9%	**0.14 (0.10, 0.19)**	**0.12 (0.09, 0.17)**	**0.69 (0.48, 0.98)**	0.85 (0.57, 1.27)	**0.22 (0.14, 0.35)**	**0.31 (0.18, 0.52)**	**0.18 (0.12, 0.29)**	**0.27 (0.17, 0.44)**
Currently has health insurance	93.0%	**0.57 (0.35, 0.91)**	**0.51 (0.31, 0.84)**	**0.36 (0.21, 0.62)**	0.58 (0.32, 1.07)	**0.40 (0.22, 0.72)**	0.73 (0.36, 1.45)	0.67 (0.32, 1.40)	1.02 (0.47, 2.18)
Had health insurance at some point in past year	95.2%	**0.47 (0.27, 0.79)**	**0.43 (0.25, 0.74)**	**0.42 (0.22, 0.81)**	0.69 (0.34, 1.39)	**0.40 (0.20, 0.77)**	0.66 (0.30, 1.44)	0.67 (0.29, 1.53)	0.97 (0.41, 2.26)

Household economic well-being									
Low household income	22.1%	**2.23 (1.67, 2.97)**	**1.88 (1.34, 2.62)**	**1.89 (1.23, 2.90)**	1.20 (0.74, 1.95)	**2.82 (1.77, 4.48)**	1.61 (0.98, 2.63)	**3.31 (2.15, 5.09)**	**2.09 (1.29, 3.41)**
Household income at or below 125% of federal poverty level^7^	15.8%	**2.07 (1.50, 2.87)**	**1.97 (1.35, 2.87)**	**2.15 (1.32, 3.50)**	1.64 (0.95, 2.80)	**2.84 (1.68, 4.80)**	**1.77 (1.05, 3.00)**	**3.03 (1.89, 4.84)**	**2.21 (1.22, 3.99)**
Has other sources of household income beyond personal earnings	19.1%	**2.02 (1.50, 2.71)**	**1.80 (1.34, 2.42)**	1.27 (0.84, 1.90)	**1.66 (1.09, 2.53)**	1.30 (0.76, 2.23)	1.53 (0.94, 2.47)	0.94 (0.52, 1.71)	0.93 (0.50, 1.74)
Currently unhoused^8^	3.7%	0.89 (0.47, 1.69)	0.76 (0.38, 1.53)	1.54 (0.48, 4.91)	1.35 (0.51, 3.56)	**4.26 (1.97, 9.19)**	**2.77 (1.32, 5.81)**	1.89 (0.82, 4.36)	1.30 (0.55, 3.08)
Experiencing financial difficulty now^9^	15.6%	1.26 (0.89, 1.78)	1.03 (0.71, 1.48)	**1.74 (1.11, 2.75)**	1.55 (0.97, 2.48)	**3.14 (1.98, 4.99)**	**2.64 (1.64, 4.24)**	**2.62 (1.60, 4.30)**	**1.88 (1.12, 3.15)**
Can sustain $400 emergency expense	76.9%	0.82 (0.61, 1.11)	0.96 (0.70, 1.32)	0.84 (0.54, 1.28)	1.05 (0.66, 1.67)	**0.57 (0.37, 0.87)**	0.79 (0.47, 1.33)	**0.41 (0.26, 0.65)**	0.63 (0.39, 1.01)
Likely to experience food, housing, or medical care hardships in next two months	16.3%	**1.58 (1.14, 2.18)**	1.36 (0.96, 1.94)	**2.02 (1.25, 3.24)**	**1.69 (1.03, 2.78)**	**2.34 (1.48, 3.71)**	**1.86 (1.13, 3.05)**	**3.37 (2.08, 5.45)**	**2.58 (1.55, 4.29)**
Received disability benefits in past year^10^	5.6%	**1.73 (1.08, 2.77)**	**1.65 (1.02, 2.67)**	1.62 (0.86, 3.05)	**1.94 (1.04, 3.62)**	0.9 (0.41, 1.98)	0.90 (0.41, 1.99)	0.93 (0.34, 2.49)	0.76 (0.28, 2.04)
Received TANF benefits in past year	1.4%	2.03 (0.83, 4.96)	1.91 (0.80, 4.59)	2.28 (0.75, 6.90)	1.92 (0.62, 5.88)	1.46 (0.50, 4.23)	1.10 (0.34, 3.52)	**4.63 (1.66, 12.95)**	**3.63 (1.37, 9.65)**
Received SNAP benefits or used food bank in past year	12.9%	**1.79 (1.29, 2.48)**	**1.83 (1.28, 2.61)**	1.01 (0.63, 1.59)	0.89 (0.53, 1.50)	**2.08 (1.27, 3.41)**	1.44 (0.85, 2.46)	**2.61 (1.60, 4.24)**	**1.80 (1.07, 3.03)**

1 Either self-employed or work for someone else as an independent contractor.

2 Category includes on-call workers, those employed by temp. agency, and those whose work is subcontracted out.

3 Contingent work is defined as a job not expected to last another 12 months.

4 App-based employment defined as jobs or tasks that connect workers to customers via an app.

5 Earnings at or below $40,000.

6 Wage theft defined as having done work for which pay not received or received less pay than expected.

7 Poverty measure calculated using household income and household size.

8 Unhoused defined by respondent not owning or renting home and stating they currently do not have permanent place to live.

9 Reports it being difficult, very difficult, or extremely difficult to live on household income now.

10 Reports receipt of SSDI, SSI, CA Disability Insurance, or Private Disability Insurance.

**TABLE 3:** Impact of independent contracting, other forms of alternative employment, contingent work, and app-based work vs. traditional forms of employment in main job on health status.

		Independent contractor^1^ Weighted percent: 16.8%		Other forms of alternative employment^2^ Weighted percent: 9.8%		Contingent work^3^ Weighted percent: 5.4%		App-based work^4^ Weighted percent: 5.6%	
Health status measures	Weighted percent with work condition	Unadjusted	Adjusted	Unadjusted	Adjusted	Unadjusted	Adjusted	Unadjusted	Adjusted
		Cells are odds ratios & 95% confidence intervals for comparison of form of employment vs. traditional. Cells in bold are statistically significant.							
Fair or poor physical health status	22.1%	1.05 (0.77, 1.45)	0.99 (0.73, 1.35)	1.09 (0.73, 1.63)	1.07 (0.71, 1.63)	0.99 (0.63, 1.54)	0.87 (0.52, 1.44)	**1.65 (1.03, 2.66)**	1.47 (0.92, 2.33)

Fair or poor mental health status	23.9%	0.78 (0.57, 1.06)	0.82 (0.60, 1.13)	0.83 (0.55, 1.26)	0.71 (0.45, 1.11)	**2.40 (1.58, 3.65)**	**1.70 (1.08, 2.68)**	**1.96 (1.26, 3.04)**	1.53 (0.94, 2.47)

High levels of perceived stress	18.8%	1.11 (0.80, 1.54)	1.15 (0.82, 1.62)	1.19 (0.77, 1.83)	1.14 (0.72, 1.81)	**2.11 (1.35, 3.29)**	1.62 (0.97, 2.70)	1.41 (0.89, 2.23)	1.09 (0.66, 1.81)

One or more chronic conditions	37.4%	1.23 (0.95, 1.60)	1.10 (0.84, 1.44)	0.76 (0.53, 1.09)	1.01 (0.69, 1.47)	1.19 (0.78, 1.83)	**1.62 (1.07, 2.45)**	1.00 (0.66, 1.54)	1.09 (0.70, 1.70)

Limited in activities	17.6%	**1.44 (1.06, 1.97)**	1.28 (0.94, 1.75)	1.15 (0.76, 1.73)	1.40 (0.92, 2.13)	1.22 (0.75, 1.99)	1.37 (0.82, 2.30)	1.40 (0.87, 2.27)	1.37 (0.86, 2.19)

Common symptoms									
Breathing problems	12.8%	1.25 (0.85, 1.83)	1.24 (0.82, 1.88)	1.54 (0.93, 2.57)	1.44 (0.88, 2.35)	1.42 (0.76, 2.67)	1.32 (0.70, 2.51)	1.38 (0.76, 2.51)	1.16 (0.61, 2.19)
Pain	23.8%	**1.52 (1.12, 2.06)**	1.33 (0.99, 1.79)	**1.49 (1.00, 2.21)**	**1.61 (1.08, 2.39)**	**1.82 (1.15, 2.88)**	**1.95 (1.24, 3.06)**	1.30 (0.79, 2.16)	1.23 (0.73, 2.06)
Numbness	19.9%	1.23 (0.88, 1.71)	1.07 (0.76, 1.51)	**1.78 (1.18, 2.67)**	**1.95 (1.29, 2.95)**	1.30 (0.77, 2.19)	1.48 (0.93, 2.36)	1.07 (0.61, 1.90)	1.13 (0.63, 2.04)

Health behaviors									
Regular exercise	78.9%	1.25 (0.88, 1.76)	1.31 (0.92, 1.86)	1.11 (0.72, 1.70)	1.08 (0.72, 1.64)	0.77 (0.47, 1.24)	0.81 (0.49, 1.33)	0.71 (0.43, 1.17)	0.72 (0.43, 1.20)
High levels of alcohol consumption^5^	14.9%	1.00 (0.72, 1.40)	1.01 (0.71, 1.43)	**1.83 (1.14, 2.94)**	**1.61 (1.00, 2.61)**	0.66 (0.33, 1.34)	0.65 (0.32, 1.33)	1.23 (0.72, 2.13)	1.31 (0.73, 2.38)
Current smoker	8.5%	**1.72 (1.17, 2.53)**	1.37 (0.89, 2.11)	**2.20 (1.16, 4.17)**	**1.86 (1.01, 3.41)**	0.98 (0.46, 2.10)	0.85 (0.37, 1.94)	**1.96 (1.11, 3.44)**	**1.74 (1.01, 3.01)**

On-the-Job Injuries									
One or More On-the-job injuries in year before interview	8.3%	1.17 (0.72, 1.89)	1.10 (0.66, 1.83)	**2.96 (1.73, 5.09)**	**2.54 (1.48, 4.35)**	1.20 (0.66, 2.16)	1.03 (0.54, 1.97)	0.90 (0.47, 1.75)	0.73 (0.35, 1.51)
Received medical treatment for injury	4.0%	0.96 (0.51, 1.79)	0.81 (0.39, 1.67)	**3.89 (2.04, 7.41)**	**3.91 (2.09, 7.33)**	0.91 (0.40, 2.09)	0.97 (0.39, 2.42)	1.39 (0.65, 2.97)	1.51 (0.66, 3.45)
Reported injury to employer	4.9%	0.79 (0.44, 1.40)	0.67 (0.36, 1.25)	**2.97 (1.6, 5.52)**	**2.64 (1.43, 4.86)**	1.14 (0.58, 2.25)	1.02 (0.49, 2.16)	0.94 (0.43, 2.06)	0.82 (0.35, 1.89)
Received workers’ compensation	1.4%	0.25 (0.06, 1.07)	**0.20 (0.04, 0.99)**	2.62 (0.83, 8.21)	1.98 (0.73, 5.36)	0.75 (0.17, 3.24)	0.47 (0.08, 2.75)	1.19 (0.35, 4.07)	1.15 (0.28, 4.65)

Medical care utilization									
Medical visits in past year	78.4%	1.08 (0.79, 1.48)	1.1 (0.79, 1.54)	**0.56 (0.37, 0.84)**	0.71 (0.46, 1.09)	0.70 (0.43, 1.14)	0.90 (0.55, 1.48)	1.41 (0.81, 2.46)	1.73 (0.95, 3.16)
Hospital admissions in past year	6.9%	**1.72 (1.14, 2.60)**	**1.6 (1.04, 2.47)**	**1.98 (1.13, 3.48)**	**1.95 (1.08, 3.52)**	0.95 (0.51, 1.77)	0.95 (0.48, 1.85)	1.50 (0.76, 2.99)	1.60 (0.80, 3.24)

1 Either self-employed or work for someone else as an independent contractor.

2 Category includes on-call workers, those employed by temp. agency, and those whose work is subcontracted out.

3 Contingent work is defined as a job not expected to last another 12 months.

4 App-based employment defined as jobs or tasks that connect workers to customers via an app.

5 Consumed more than five drinks in one sitting on two or more occasions in the past month.

**TABLE 4:** Working conditions of workers in independent contracting, other forms of alternative employment, contingent work, and app-based work vs. traditional forms of employment in main job.

		Independent contractor^1^ Weighted percent: 16.8%		Other forms of alternative employment^2^ Weighted percent: 9.8%		Contingent work^3^ Weighted percent: 5.4%		App-based work^4^ Weighted percent: 5.6%	
Working conditions	Weighted percent with work condition	Unadjusted	Adjusted	Unadjusted	Adjusted	Unadjusted	Adjusted	Unadjusted	Adjusted
		Cells are odds ratios & 95% confidence intervals for comparison of form of employment vs. traditional. Cells in bold are statistically significant.							
Current job									
Flexible hours	58.2%	**3.99 (2.90, 5.47)**	**5.13 (3.72, 7.07)**	0.90 (0.63, 1.29)	0.91 (0.61, 1.36)	0.95 (0.62, 1.45)	0.91 (0.56, 1.49)	**1.96 (1.20, 3.19)**	**2.23 (1.26, 3.93)**
Irregular shifts	23.5%	**4.35 (3.32, 5.70)**	**4.63 (3.48, 6.16)**	**1.66 (1.13, 2.45)**	**1.79 (1.17, 2.72)**	1.39 (0.88, 2.19)	1.12 (0.70, 1.79)	**3.14 (2.08, 4.76)**	**2.23 (1.39, 3.57)**
Required overtime	51.2%	0.98 (0.76, 1.26)	0.94 (0.72, 1.22)	**1.48 (1.04, 2.11)**	1.40 (0.97, 2.01)	**0.65 (0.43, 0.98)**	0.79 (0.53, 1.17)	**0.56 (0.36, 0.87)**	0.68 (0.42, 1.12)
Work from home some or all of the time	48.7%	**1.85 (1.42, 2.40)**	**3.27 (2.40, 4.45)**	0.74 (0.52, 1.05)	1.13 (0.74, 1.73)	0.72 (0.48, 1.09)	1.04 (0.57, 1.89)	**0.66 (0.44, 0.99)**	1.25 (0.77, 2.04)
High levels of ergonomic demands^5^	27.2%	**1.74 (1.32, 2.31)**	**1.52 (1.11, 2.08)**	**2.51 (1.72, 3.66)**	**1.87 (1.22, 2.85)**	1.43 (0.92, 2.21)	1.15 (0.72, 1.86)	**1.91 (1.24, 2.93)**	1.44 (0.89, 2.35)
High levels of cognitive demands^6^	41.7%	**0.45 (0.34, 0.60)**	**0.57 (0.42, 0.77)**	**0.67 (0.46, 0.97)**	0.95 (0.63, 1.44)	0.69 (0.45, 1.06)	0.90 (0.56, 1.43)	0.63 (0.40, 1.00)	0.88 (0.54, 1.44)
Job combines high level of demands and low control^7^	15.4%	**0.53 (0.36, 0.78)**	**0.57 (0.38, 0.84)**	**1.81 (1.21, 2.73)**	**1.72 (1.15, 2.57)**	**2.12 (1.34, 3.37)**	**1.81 (1.11, 2.94)**	1.01 (0.57, 1.78)	0.70 (0.38, 1.27)
Experienced bullying by coworkers, supervisors, or customers on job	41.6%	0.82 (0.64, 1.06)	0.85 (0.65, 1.12)	1.09 (0.76, 1.57)	1.23 (0.85, 1.78)	1.27 (0.84, 1.92)	1.16 (0.75, 1.79)	1.42 (0.94, 2.14)	1.19 (0.75, 1.88)
Respectful treatment from coworkers, supervisors, or customers on job	87.2%	0.77 (0.51, 1.15)	0.89 (0.57, 1.38)	**0.51 (0.31, 0.82)**	**0.57 (0.35, 0.92)**	0.55 (0.30, 1.00)	0.62 (0.33, 1.15)	0.72 (0.43, 1.22)	0.94 (0.54, 1.63)
Job requires less education than worker has^8^	25.5%	0.89 (0.67, 1.17)	0.80 (0.60, 1.06)	0.71 (0.50, 1.02)	0.71 (0.48, 1.04)	**1.59 (1.03, 2.45)**	1.35 (0.88, 2.06)	**1.62 (1.07, 2.45)**	1.15 (0.74, 1.78)
Covered by union or employee association contract	21.0%	**0.40 (0.27, 0.59)**	**0.35 (0.23, 0.53)**	0.80 (0.53, 1.21)	0.87 (0.57, 1.34)	**0.56 (0.32, 0.99)**	0.82 (0.44, 1.55)	**0.32 (0.16, 0.62)**	**0.39 (0.19, 0.84)**

**Career**									
New better job or promotion in current job in past year	30.8%	**0.52 (0.39, 0.70)**	**0.62 (0.46, 0.83)**	1.28 (0.87, 1.87)	1.12 (0.75, 1.67)	**0.59 (0.36, 0.95)**	**0.37 (0.23, 0.59)**	0.69 (0.43, 1.10)	**0.54 (0.34, 0.88)**
Experience of discrimination in past^9^	7.9%	**1.27 (0.87, 1.86)**	1.45 (0.98, 2.16)	1.32 (0.72, 2.44)	1.35 (0.77, 2.38)	**3.97 (2.46, 6.42)**	**3.24 (1.92, 5.47)**	1.26 (0.68, 2.37)	0.97 (0.50, 1.88)

1 Either self-employed or work for someone else as an independent contractor.

2 Category includes on-call workers, those employed by temp. agency, and those whose work is subcontracted out.

3 Contingent work is defined as a job not expected to last another 12 months.

4 App-based employment defined as jobs or tasks that connect workers to customers via an app.

5 High ergonomic demands based on sum of a list of physical demands done some or a lot, then dichotomized at 75th percentile or higher. List includes walking, use of stairs, standing for 4 or more hours a shift, sitting for long periods, stooping, crouching or kneeling, bending or twisting, carrying 10 or 50 lbs., pinching or grabbing, using vibrating tools, and riding in a vehicle.

6 High cognitive demands based on sum of list of cognitive demands done some or a lot, then dichotomized at 75th percentile or higher. List includes use of computer or other electronic devices, concentration for long periods, and interacting with other people.

7 Combination of high job demands and low control over job.

8 Respondents’ response to item about education level required to do job compared to their reported level of education.

9 Respondents’ report of having been fired, not hired, or not receiving a promotion on basis of age, sex, gender identity, sexual orientation, race or ethnic background, and/or disability.
